# Attitudes towards air quality during outdoor exercise amongst habitual exercisers

**DOI:** 10.1002/ejsc.12194

**Published:** 2024-10-24

**Authors:** Scarlett Moloney, Jane M Black, Valerie Gladwell, Nic Bury, Gavin Devereux

**Affiliations:** ^1^ School of Allied Health Sciences University of Suffolk Ipswich UK; ^2^ School of Psychology and Sport and Sensory Sciences Cambridge Centre for Sport and Exercise Sciences Anglia Ruskin University Cambridge UK; ^3^ Institute of Health and Wellbeing Research University of Suffolk Ipswich UK; ^4^ Institute for Life Sciences University of Southampton Southampton UK

**Keywords:** air pollution, attitudes, exercise, health, physical activity

## Abstract

The effects of air pollution on health outcomes are well‐established. However, little is known about perceptions of air pollution and how it may influence exercise behaviours. The aim of this study was to understand the perceived importance of air pollution during planned exercise, and where relevant, explore how those perceptions may differ between those living in different locations. A questionnaire was disseminated to several running and cycling clubs in the United Kingdom, covering population characteristics to determine urban, rural or coastal residents and exercisers, self‐reported asthma, perceptions of air quality during active travel, planned exercise and attitudes towards learning more about the topic. Anonymised responses were gained from 381 adult participants (≥18 years and 60% female), 119 of whom answered questions related to active travel. More than half (54%) of all respondents think about the air quality they are exposed to during exercise and how it may affect their health. More urban than rural respondents (59% vs. 47% and *p* = 0.03) think about air quality and the impact it may have on their health when exercising. There were insufficient coastal respondents for direct comparison. Most survey respondents (57%) state that they would take the most severe course of action regarding exercise behaviour by avoiding it altogether during periods of heavy road traffic. Individuals with self‐reported asthma (*n* = 60), irrespective of place of residence, are the most likely to be conscious of the potential effects of air pollution on their health and exercise performance compared to counterparts without self‐reported asthma.

## Highlights

- Most survey respondents (57%) stated that they would avoid outdoor exercise altogether if they perceived the road traffic volume to be high.
- More urban than rural residents (59% vs. 47% and *P* = 0.03) think about how the air quality that they are exposed to during outdoor exercise may affect their health.
- Individuals with self‐reported asthma, irrespective of place of residence, are the most likely to be conscious of the potential effects of air pollution on their health and exercise performance.
- Regardless of location, the majority of all participants (69%) would like to learn more about air quality and the impact it could have on their health during their exercise routines.

## 1 INTRODUCTION

The adverse impact of air pollution exposure on health outcomes and the development of non‐communicable diseases are reasonably well‐established (Al‐Kindi et al., 2020; Badulescu et al., 2019; Holgate, 2022). However, little is known about peoples' perceptions of air pollution, the importance they place on the topic of air quality and how it may (if at all) influence their exercise behaviours (Tainio et al., 2021).

Due to the increased minute ventilation that occurs during exercise, the volume of inhaled air pollutants is amplified (Oravisjärvi et al., 2011), and therefore, the associated health risks may be exacerbated (Giles et al., 2014; Kim et al., 2015; Thurston et al., 2017). A complex interplay of risk factors may modulate the combined effects of air pollution and exercise on health outcomes including pollution exposure, exercise intensity and pre‐existing health conditions (Qin et al., 2019). Physical activity and planned exercise are regularly promoted for the well‐known benefits of preventing non‐communicable diseases and improving mental health (Klaperski et al., 2019; Nystoriak et al., 2018; Vina et al., 2012). With the World Health Organisation's global initiative to reduce physical inactivity by 15% by 2030 (Bull et al., 2020), factors that may discourage individuals from participating in physical activity and planned exercise should be considered and managed where possible. Given that air pollution is one of the largest environmental risks to health (Fuller et al., 2022), media coverage, peoples' developing knowledge of this topic and the associated perceptions that arise could deter individuals from physical activity and exercise participation.

There is some emerging evidence to support this, as a recent mapping review found that on days where air pollution is high, individuals are less likely to engage in physical activity and that highly polluted environments may inhibit people from performing physical activity overall (Tainio et al., 2021). A meta‐analysis investigating the effect of air pollution on physical activity behaviours in adults concluded that the likelihood of avoiding physical activity was increased by 1.1% per one unit increase in PM_2.5_ exposure, and more generally, all included studies reported increasing air pollution concentrations to be negatively associated with physical activity (An et al., 2018). Accordingly, there appears to be a relationship between a decrease in overall air quality and reduced levels of physical activity (An et al., 2019; Smith et al., 2019). Previously, Wen and colleagues (Wen et al., 2009) reported that participants changed or reduced time spent being physically active outdoors in response to media alerts about the air quality index (AQI) and this was more common for participants with asthma. This may suggest that those with asthma may have an increased awareness of the potential health impact of ambient air pollution compared to those without asthma (Wen et al., 2009). Separately, it is known that air pollution exposure can exacerbate asthma symptoms and even contribute to the development of new‐onset asthma (Guarnieri et al., 2014).

Whilst these findings are valuable, it is important to highlight that existing research has considered the impact of pollution exposure on physical activity behaviours but not specifically on planned exercise routines. The differentiation of physical activity and planned exercise may be important because physical activity is often spontaneous, and the location where it is performed is therefore determined by convenience and coincidence. This is unlike planned exercise where it may be perceived that there is more freedom to select a location or route Thus, air quality or perceptions of air quality may be a greater consideration for and have a greater influence on planned exercise behaviours (Fisher et al., 2016). For these reasons, this study will focus on an adult population only as children often do not have autonomy when deciding on exercise routes.

Up to now, urban environments have received the most attention when considering air pollution, exercise and health (Hodgson et al., 2018; Pasqua et al., 2020), as it is widely expected that greater levels of air pollution will materialise in them compared to other locations and the types or species of pollutants found in heavily urbanised areas will be the most harmful to health (Barman et al., 2010). Therefore, it would be of interest to investigate whether exercisers who perceive that they live in an urban location have a greater consideration for air pollution and its potential effects, and where possible, to explore potential differences between those with and without self‐reported asthma. Therefore, the aim of this study is to understand the perceived importance of air pollution during planned exercise amongst adult habitual exercisers, and where relevant, explore how those perceptions may differ between those living and exercising in different locations.

## 2 METHODS

### 2.1 Questionnaire design

This questionnaire was designed to explore the attitudes and perceptions towards air quality during exercise in habitual outdoor exercisers. Titled ‘The Perceived Importance of Air Quality During Outdoor Exercise’ (see Supporting Information S1), the online questionnaire was developed on Microsoft Forms for anonymous responses to be collected. Members of a local running club with and without self‐reported asthma were consulted to inform the design and phrasing of the questionnaire. Also, university students and staff who regularly exercise outdoors were included in pilot testing that informed adjustments to the language and phrasing used within the questionnaire to ensure face validity (Boparai et al., 2019). Quantitative data were collected via closed‐ended questions and Likert scale responses. The first section of the questionnaire covered population characteristics via closed‐ended questions. These questions provided nominal data to inform the environment in which the participant lived and exercised in (urban, rural or coastal) and whether participants self‐reported asthma. In the following sections of the questionnaire, perceptions of air quality during active travel, planned exercise and attitudes towards learning more about the topic were explored using questions with a 5‐point Likert scale response to provide ordinal data. For each statement, participants were instructed to select how strongly they agreed or disagreed by choosing the most appropriate answer (strongly disagree, disagree, neither agree nor disagree, agree and strongly agree). Only participants who routinely run, walk, or cycle to their place of work were given the option to answer the active travel section of the questionnaire.

To reduce acquiescence bias, some statements were framed positively and others negatively to ensure internal validity (Boparai et al., 2019; McDermott, 2011). Central tendency bias is a recognised limitation of Likert scale questionnaires, so statements were written clearly and concisely in a non‐scientific language to better enable participants to understand the terminology in the statement. This should have reduced the likelihood of the central or neutral answer being chosen due to lack of understanding (Boparai et al., 2019).

The study and the questionnaire used within it was approved by the University's Research Ethics Committee (RETH(P)21/016).

### 2.2 Data collection

A purposive and subsequent snowball sampling method was used to recruit participants. To be eligible to take part in the questionnaire, participants had to routinely engage in at least one planned outdoor exercise session per week. A link to the online questionnaire was disseminated directly by emailing numerous running and cycling clubs in the United Kingdom and indirectly by posting on the social media platforms of numerous running and cycling clubs. Anonymised responses were gained from 381 participants, 119 of whom answered questions related to active travel, between 7th April and the May 30, 2022. On average, the questionnaire took 4 min and 6 s to complete and by submitting the online form, consent to use anonymised responses for the purpose of the study was implied.

### 2.3 Data analysis

There were two phases to data analysis to provide both descriptive and inferential statistics. Firstly, raw data were transferred to Microsoft Excel to determine the descriptive statistics of nominal categories to describe the participants (percentages, frequencies and mode). Following this, the data were exported to SPSS for inferential statistical analysis. Likert scale responses were coded from 1–5, whereby 1 = strongly disagree and 5 = strongly agree, as were presented on the questionnaire to participants; similarly, responses to questions that provided nominal data were also numerically coded for the purpose of statistical analysis.

A Kruskal–Wallis one‐way analysis of variance test was used to detect significant differences in responses between participants who perceived that they live and exercise in an urban location and participants who perceived that they live and exercise in a rural location. Only 7% of participants perceived that they live and exercise in a coastal location; therefore, participants from this group were not included in the analysis. The test was conducted to test for significant differences in responses for two key sections including (i) perceptions of air quality during planned exercise and (ii) attitudes towards learning more about the impact of air pollution during exercise on health. For all statistical tests, a *p*‐value of 0.05 or less was considered statistically significant.

## 3 RESULTS

### 3.1 Characteristics of respondents

Descriptive statistics were used to report participant characteristics in the form of percentages (Table 1). A total of 381 participants completed the questionnaire, with an almost equal response rate from those who perceive their location as urban and rural, although the coastal response rate was far lower. The 16% rate of self‐reported asthma diagnosis is slightly greater than the official estimate of 12% of the UK national population diagnosed with asthma (Mukherjee et al., 2014). Almost half of the participants reported that on average they engaged in 4 h or more of outdoor exercise per week, with the lower volumes of planned exercise being far less common. Only 31% of participants who stated that they engaged in active travel (run, walk and/or cycle) at least 3 times per week answered the Likert scale questions relating to perceptions of air quality during active travel.

**TABLE 1:** Participant characteristics and planned exercise behaviours.

Participant characteristics	Number of participants (%)
Gender	
Male	150 (39%)
Female	231 (60%)
Prefer not to say	1 (<1%)
Self‐reported asthma	
Yes	60 (16%)
No	321 (84%)
Location	
Urban	184 (48%)
Rural	171 (45%)
Coastal	27 (7%)
Hours spent exercising per week	
Up to 1 h	7 (2%)
1 up to 2 h	33 (9%)
2 up to 3 h	65 (17%)
3 up to 4 h	97 (25%)
More than 4 h	180 (47%)
Type(s) of outdoor exercise	
Running	297 (46%)
Walking	261 (40%)
Cycling	93 (14%)
Active travel	119 (31%)
Walk	84 (45%)
Run	53 (28%)
Cycle	49 (26%)

### 3.2 Planned exercise

**FIGURE 1:**
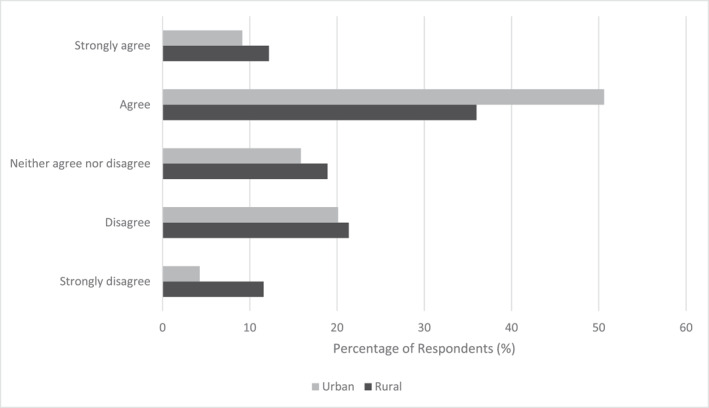
Responses to ‘I think about how the air quality that I am exposed to during exercise may affect my health.’ Responses between rural and urban participants were significantly different (*p* = 0.03).

Participants were asked to select how strongly they agreed or disagreed with statements relating to the perceptions of air quality during planned exercise. Most participants (54%) agreed or strongly agreed ‘I think about how the air quality that I am exposed to during exercise may affect their health’, whilst 18% neither agreed nor disagreed and 28% disagreed or strongly disagreed. Responses between rural and urban participants were significantly different (*p* = 0.03). The most common response for both groups was ‘agree’; 47% of rural and 59% of urban respondents, respectively (Figure 1). Interestingly, when considering responses from those with and without self‐reported asthma separately, a greater percentage of those with self‐reported asthma (67%) compared to participants without self‐reported asthma (52%) agreed or strongly agreed with this same statement. Accordingly, a greater percentage of participants without self‐reported asthma (29%) compared to those with self‐reported asthma (19%) disagreed or strongly disagreed with the statement.

Responses to a closed‐ended question, ‘does the volume of road traffic influence your exercise routine?’ revealed that 57% of respondents avoid exercise when there is a perceived high volume of traffic, whereas 43% selected ‘traffic volume does not influence my exercise routine’. Furthermore, 7% of respondents check the air quality forecast in the location they are planning to exercise in. Respondents who selected that they do check the air quality forecast for the location they plan to exercise in were asked further closed‐ended questions on this topic. In response to ‘how does the air quality forecast influence your choice to exercise?’, 28% would refrain from exercise if the air quality was classified as ‘poor’; 28% selected that they were more likely to exercise outdoor if the air quality is classified as ‘good’; 32% would consider an alternative location where the air quality is better according to the forecast and 12% of respondents reported that this does not influence their exercise routine in any way.

Responses from all participants were least consistent to the final statement, ‘I think my ability to exercise is affected by the air pollution I am exposed to’, with 35% of participants disagreeing or strongly disagreeing, 35% agreeing or strongly agreeing and 30% neither agreeing nor disagreeing. Whilst responses between urban and rural respondents were not different (*p* = 0.14), most respondents with asthma (60%) compared to without asthma (33%) agreed or strongly agreed with the statement.

### 3.3 Active travel

**FIGURE 2:**
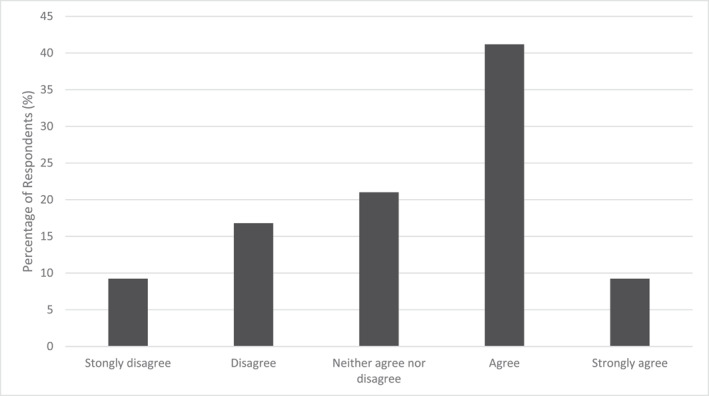
Responses to ‘I think about how the air quality that I am exposed to when I travel to and from work may affect my health’.

One hundred nineteen of the participants run, walk or cycle to their place of work. When asked to consider active travel separately, over half (53%) of those respondents agreed or strongly agreed to ‘I think about how the air quality that I am exposed to when I travel to and from work may affect my health’, whilst 28% disagreed or strongly disagreed and 21% neither agreed nor disagreed (Figure 2). There was a larger disparity in responses to ‘I do not think about air quality when deciding upon the route I take to get to and from work’, as 46% of respondents agreed or strongly agreed, 36% disagreed or strongly disagreed and 18% neither agreed nor disagreed. Due to there being nearly double the number of urban compared to rural respondents for the active travel section, it was not appropriate to use inferential analysis to assess for statistical differences between locations.

### 3.4 Current knowledge and attitudes towards learning about air pollution

**FIGURE 3:**
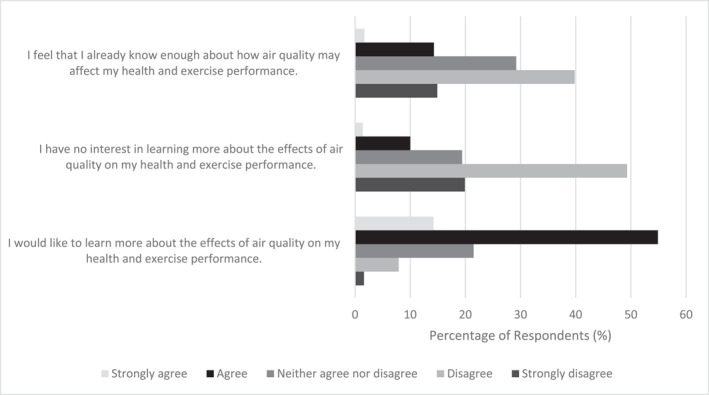
Current knowledge and attitudes towards learning about air pollution.

The final section of the questionnaire aimed to investigate attitudes towards learning more about air quality and exercise. This revealed that 69% of respondents agreed or strongly agreed to ‘I would like to learn more about the effects of air quality on my health and exercise performance’, whilst only 8% disagreed and 22% neither agreed nor disagreed. To check for acquiescence bias, 69% of respondents disagreed or strongly disagreed that ‘I have no interest in learning more about the effects of air quality on my health and exercise performance’, 11% agreed or strongly agreed with the statement and 19% neither agreed nor disagreed. Regarding perceptions of current knowledge of the topic, over half of respondents (55%) disagreed or strongly disagreed in response to ‘I feel that I already know enough about how air quality may affect my health and exercise performance’, whereas 16% agreed or strongly agreed and 29% neither agreed nor disagreed. Figure 3 summarises these data.

## 4 DISCUSSION

The findings of this study show that air quality and pollution is an important consideration for the typical habitual exerciser in all locations. More than half (54%) of all respondents think about the air quality they are exposed to during exercise and how it may affect their health. Following a review of all 381 responses, analysis was concentrated on urban (*n* = 183) and rural environments (*n* = 171), and a trend in the analysis shows that those identifying as urban residents and habitual exercisers may be particularly conscious of the potential implications of exercising in a polluted environment. For example, when responses are differentiated by location, more urban (59%) than rural (47%) respondents think about the air quality and the impact it may have on their health when exercising. As the response rate for coastal (*n* = 27, 7%) was deemed too low to include in comparative analysis, we will comment on the responses and findings from those identifying as coastal residents separately.

Despite air quality and pollution being a consideration during planned exercise for most of the respondents, interestingly, only a very small minority (7%) actively check air quality information before setting out to exercise. This is despite a far greater proportion of all respondents stating that they would either alter their planned exercise in some form (up to 32%) or avoid it altogether (28%) if they knew air pollution levels were high. Therefore, there appears to be a disparity between the perceived importance of air quality individuals are exposed to during exercise and the act of finding objective information about it. There exist national air quality indexes across the world, and here in the United Kingdom, we have the Daily AQI as part of the UK Air Information Resource (DEFRA). This information is often provided in widely used weather forecast apps on various Internet sites and mobile software platforms, so arguably, it is easily accessible. It may be that the AQI information provided is not viewed and considered amongst the other climatic information presented and/or that the air quality information is not readily understood. More research may be needed to better understand these factors.

Another interesting finding from this study is that most respondents avoid exercising during periods of peak road traffic. Regarding the perceived importance of road traffic volume, it is likely not appropriate or valid to directly compare urban and rural (and coastal) respondents, given that road traffic is more likely to be an urban topic of interest. However, we may look at urban responses alone. For those living in urban environments, the prevalence of traffic related air pollution news reports (Kenis et al., 2022) may be of more interest compared to those identifying as living and exercising in a rural environment. Initially, reviewing all responses collectively, the majority of all survey respondents (57%) state that they would take the most severe course of action regarding exercise behaviour, by avoiding it altogether during periods of heavy road traffic. This could be particularly worrisome given that 82.9% of the population in England (DEFRA, 2021), and more broadly, 56% of the global population (The World Bank, 2023) are known to live in an urban environment, and this is projected to increase further in the future, with 7 out of 10 people projected to live in cities by the year 2050 (The World Bank, 2023). Perceived barriers to exercise are known to be strongly associated with physical inactivity across the lifespan (Chen, 2010; Langguth et al., 2015; Nooijen et al., 2018) and subsequently the prevalence of non‐communicable diseases throughout our lifetime (Katzmarzyk et al., 2022; Lake et al., 2009; Liese et al., 2013).

Separate to the comparison of those identifying as urban and rural residents and exercisers, there appears to be a consistent trend in the questionnaire responses for those individuals self‐reporting an asthma diagnosis. These individuals, irrespective of place of residence and habitual exercise, are the most likely to be conscious of the potential effects of air pollution on their health and exercise performance. As previously discussed, 54% of all respondents think about air quality and their health when exercising, this increases even further to 67% of those self‐reporting an asthma diagnosis, and these same respondents are also more likely to avoid exercise during periods of peak road traffic; 64% of respondents with self‐reported asthma compared to 57% of all respondents combined. Asthma is an obstructive airway disease characterised by bronchial hyperresponsiveness and inflammation of the respiratory tract (Tiotiu et al., 2020), and air pollution has been shown to elicit exacerbations in pre‐existing asthma (Guarnieri et al., 2014; Kim et al., 2013; Weinmayr et al., 2010). The oxidative stress induced by pollutant inhalation is suggested to be a key mechanistic factor for the subsequent epithelial cell inflammation and airway hyperreactivity, caused by the recruitment of neutrophils to the airways to produce reactive oxygen species (Tiotiu et al., 2020). Exposure to environments with a high particulate matter concentration for even a transient period leads to a greater fractional deposition of ultrafine particles in individuals with self‐reported asthma compared to those without (Bosson et al., 2008). Therefore, the heightened awareness amongst individuals with self‐reported asthma found in this study is relatively easily understood, as there are well‐known physiological mechanisms responsible for increased sensitivity to air pollution.

Regarding active travel, it was perhaps unsurprising that the 119 responses for that section of the questionnaire were dominated by those identifying as living in an urban location, given the likely closer proximity to the workplace for those individuals and therefore the viable option of active travel to and from work. Whilst the majority was more modest on this occasion at 53%, it was interesting to note that more people are conscious of air pollution when undertaking active travel than not. Given the two previous points, this is perhaps connected with the previously discussed point that whilst the majority of all respondents are conscious of air pollution associated with planned exercise, significantly more urban respondents are compared to their rural counterparts. Given that active travel can play a meaningful role in accumulating the desired volume of exercise for improved health outcomes (Buehler et al., 2019; Laverty et al., 2013; Song et al., 2013), this finding may be of interest for the varied groups involved in the technical and political processes of urban planning as well as those in public health. With the built, urban environment, the topic of the lifespan is again likely very important, given that active travel to and from school (van Sluijs et al., 2009) is equally relevant as the workplace for many in the urban population (Laverty et al., 2013; Song et al., 2013), although this was not a focus of this study.

As previously mentioned, it was not appropriate to include those respondents identifying as coastal residents and exercisers in comparative analyses. Regarding these coastal residents and exercisers, it was interesting to note that they generally followed the response patterns of the rural participants. Whilst we do not directly make comparisons against urban respondents, this does further support the notion that those identifying as urban residents and exercisers are particularly conscious of and reactive to perceived air quality and pollution levels when planning exercise. The final key finding of this study is that a meaningful majority of respondents (69%) wish to learn more about the effects of air quality on health and exercise performance. There were no differences between urban and rural locations for this point. This highlights the perceived importance of this topic amongst the types of habitual exercisers surveyed in this study and the need to continue to research and disseminate knowledge in an effective manner to the public. Future work could explore some of these points raised using qualitative methods, such as interviews or focus groups, to gain a greater insight into how air quality influences exercise choices.

### 4.1 Limitations

Various types of bias could have impacted the validity of the current study. For example, it is likely that habitual exercisers who have a greater awareness and perhaps place greater importance on air pollution were more likely to engage with and complete this questionnaire. Therefore, the responses may not be representative of all habitual exercisers. However, there were a range of answers for all questions and the responses do not suggest skewed or significant bias. Another limitation of the study was there not being a statistically sufficient number of respondents who perceived they live and exercise in a coastal location, to include the subgroup in the comparative analysis. Lastly, we did not explore some demographic detail such as socioeconomic status and the social gradient of health inequalities; the influence these factors may have on attitudes towards exercise and air pollution should be explored in future work.

### 4.2 Conclusion

In summary, the findings of this study show that air quality and pollution is an important consideration for habitual exercisers R. C. Murthy in urban, rural and coastal locations. Both urban residents and exercisers and individuals with self‐reported asthma appear to be particularly aware of air pollution and the effects it may have on their health. Of note, a meaningful number of all respondents would avoid exercise altogether if they knew of or perceived poor air quality.

## CONFLICT OF INTEREST STATEMENT

The authors have no competing interests to declare that are relevant to the content of this article.

## Supporting information

Supporting Information S1
