# Supplementary material for: Attitudes towards air quality during outdoor exercise amongst habitual exercisers
Source: Eur J Sport Sci. 2024 Oct 24;24(11):1671–9. doi: 10.1002/ejsc.12194 (PMC11534648; doi:10.1002/ejsc.12194)
Supplement: Supplementary file 1 — Supporting Information S1 [file EJSC-24-1671-s001.docx]

Attitudes towards Air Quality during Outdoor Exercise.

Part A)

1. Which gender do you normally identify as?
2. How would you describe the location where you live?
3. Are you diagnosed with Asthma?
4. On average, how many hours do you spend exercising outdoors per week at the moment?
5. Do you routinely (at least 3 times per week) walk/run/cycle to and from your place of work?
6. Which form(s) of active travel do you routinely use?

Part B)

**Active travel**

1. How strongly do you agree or disagree with the following statements?

I think about how the air quality that I am exposed to when I travel to and from work may affect my health.

I do not think about air quality when deciding upon the route I take to get to and from work.

**Planned Exercise (not including active travel)**

1. Which form(s) of planned outdoor exercise do you participate in weekly?
2. How strongly do you agree or disagree with the following statements?

Separate to exercise, I think about the air quality in the area that I live in.

I think about how the air quality that I am exposed to during exercise may affect my health.

I do not think about air quality when deciding upon an exercise route.

I think my ability to exercise is affected by the air pollution I am exposed to.

Part C)

**Route choices**

1. How is your exercise route influenced by your feeling of safety?
2. Does the volume of road traffic influence your exercise routine?
3. Do you check the air quality forecast for the location you are planning to exercise in?
4. How does the air quality forecast influence your choice to exercise?

Part D)

**Attitudes towards learning more about air quality**

1. How strongly do you agree or disagree with the following statements?

I would like to learn more about the effects of air quality on my health and exercise performance.

I have no interest in learning more about the effects of air quality on my health and exercise performance.

I feel that I already know enough about how air quality may affect my health and exercise performance.
